# How Information Exposure Shapes Risk Perceptions and Vaccination Intentions Among Gay, Bisexual, and Other Men Who Have Sex With Men: Cross-Sectional Survey Study

**DOI:** 10.2196/70635

**Published:** 2025-06-18

**Authors:** Doug Cheung, Luyao Xie, Lijuan Wang, Siyu Chen, Xinge Li, Zheng Zhang, Xinyue Chen, Shen Ge, Fuk-yuen Yu, Yuan Fang, Zihuang Chen, Zhennan Li, Fenghua Sun, Phoenix Mo, Yingjie Liu, Zixin Wang

**Affiliations:** 1 Centre for Health Behaviours Research JC School of Public Health and Primary Care The Chinese University of Hong Kong Hong Kong China (Hong Kong); 2 Beijing Chaoyang District Center for Disease Control and Prevention Beijing China; 3 Department of Health and Physical Education, the Education University of Hong Kong Hong Kong China (Hong Kong); 4 Danlan Goodness, Beijing, China Beijing China; 5 Hongshiliu Goodness Beijing China

**Keywords:** mpox, gay, bisexual, and other men who have sex with men, GBMSM, multigroup structural equation modeling

## Abstract

**Background:**

Exposure to information about mpox may shape distinct perceptual processes that influence vaccination intent. Understanding how such information impacts perceived risk and vaccination intention is crucial for designing effective risk communication and public health messaging, particularly among populations at high risk such as gay, bisexual, and other men who have sex with men (GBMSM).

**Objective:**

This study examined the specific pathways through which mpox information exposure and associated perceptual processes influence vaccination intent. Differences between GBMSM in Beijing and Hong Kong were also examined to explore potential contextual influences.

**Methods:**

We conducted a cross-sectional survey of mpox-unvaccinated GBMSM in Hong Kong (n=470) and Beijing (n=519) between November 2023 and March 2024. Structural equation modeling was conducted to estimate the direct and indirect effects of information exposure, perceptual processes (eg, perceived control and threat perceptions), and perceived risk on vaccination intent. Multigroup structural equation modeling was used to estimate the effect measure modification by city.

**Results:**

Exposure to positive mpox information significantly enhanced perceived control (β=0.33; *P*=.001) and increased vaccination intention through heightened perceived risk of contracting mpox (β=0.27; *P*<.001) in the following 6 months. The indirect effect of positive information exposure on vaccination intent via perceived control and risk was significant in Hong Kong (*B*=0.01; Wald test: *Z*=3.05 and *P*=.002) but not in Beijing (*B*=−0.01; *P*=.25). Conversely, negative information exposure primarily increased threat perceptions (Hong Kong: *B*=0.33 and *P*=.001; Beijing: *B*=0.93 and *P*<.001) but did not consistently translate to increased perceived risk of contracting mpox (Hong Kong: *B*=−0.10 and *P*=.31; Beijing: *B*=0.20 and *P*=.11). Notable contextual differences emerged between Beijing and Hong Kong—participants in Beijing reported higher levels of information exposure (eg, international mpox statistics; mean score 2.17, SD 0.97 vs 1.79, SD 0.89; *P*<.001) and more nonregular sex partners (mean 2.20, SD 6.73 vs 1.65, SD 3.37; *P*=.02), but the associations among information exposure, perceived risk, and vaccination intent were weaker than in Hong Kong participants.

**Conclusions:**

Positive mpox-related information strongly promotes vaccination intent by enhancing perceived control and amplifying perceived risk, particularly in settings with accessible vaccination programs such as Hong Kong. Conversely, negative information appears less effective in driving vaccination intention. Tailored, stigma-free communication is crucial for improving vaccination uptake, especially in mainland China, where subsidized vaccines are scarce and perceptual pathways linking information exposure to vaccination intent are relatively weak. Enhancing access to vaccines and addressing contextual barriers can further optimize the impact of such interventions.

## Introduction

### Background

The 2022 multicountry mpox outbreak spread rapidly via air travel and undetected local transmissions among gay, bisexual, and other men who have sex with men (GBMSM) driven by social stigma, marginalization, and discrimination [1-5]. These factors often compel GBMSM to seek anonymous and multiple same-sex connections in high-risk environments, facilitating “superspreader” events and exacerbating both local and global mpox transmission [2,5-8]. The mpox outbreak exemplifies the critical importance of a One Health approach by revealing the fundamental interconnectedness or existential disconnectedness among human, animal, and environmental health. Of concern, originally a zoonotic disease with animal-human transmission in parts of Africa, mpox has adapted to “human-to-human” transmission networks worldwide, particularly in urban environments and initially mostly affecting GBMSM. This evolution demonstrates how ecological shifts, human mobility, and social determinants—including stigma and health care inequities—collectively shape infectious disease dynamics [2]. In 2023, Asia experienced sustained imported mpox cases, with mainland China documenting 501 cases in August alone [9-11]. The region faces unique challenges due to persistent stigmatization of same-sex behaviors, limited mpox vaccination programs, and the easing of COVID-19 travel restrictions [12,13], raising concerns about a resurgence if vaccine uptake among GBMSM remains low [4,6,14].

While Western countries effectively curtailed the outbreak through culturally sensitive campaigns—such as leveraging gay-identifying physicians and influencers to boost vaccine acceptance [15,16]—Asian contexts have struggled [17]. Despite the US Centers for Disease Control and Prevention recommending the 2-dose JYNNEOS vaccine for high-risk GBMSM [18], costs and access remain significant barriers. For example, JYNNEOS costs up to US $300 per dose in higher-income settings, whereas Hong Kong has offered fully subsidized vaccination since October 2022. However, this program is largely inaccessible to mainland Chinese GBMSM, and mpox-specific vaccines are still under development [19-21]. Even in places with subsidized programs such as Hong Kong and Taiwan, vaccine uptake remains suboptimal (21.7% in Hong Kong [22] and 59.9% in Taiwan [23], much lower than in Western cities such as Paris at 78% [24]) and below the modeled 80% threshold needed to prevent further outbreaks [25].

A growing body of research has examined risk perception and media exposure among GBMSM [22,26-28], but few studies have explored how varying types and sources of mpox-related information shape illness perceptions and vaccination intentions—especially in Chinese contexts. Most notably, the psychological mechanisms connecting information exposure with preventive intentions and actions remain poorly understood in Asia, where negative media portrayals and lesbian, gay, bisexual, transgender, and queer (LGBTQ+) stigma are widespread [29-31]. This constitutes a critical research gap as understanding these mechanisms is essential for designing effective, culturally sensitive risk communication strategies.

The common sense model of illness self-regulation (CSM) provides a valuable framework for examining how different types of mpox-related information influence GBMSM’s preventive intentions and behaviors. The extended CSM framework proposes that emotional states such as concerns, depression, and distress are more strongly correlated with threat-related illness perceptions, including the identification of symptom severity, duration, and their impacts on individuals [32]. For example, exposure to stigmatizing news about deaths due to mpox and HIV or AIDS coinfection may evoke negative emotions and elevate GBMSM’s perceptions of mpox severity and impact, leading to maladaptive coping responses such as denial, avoidance, or delayed treatment seeking [32,33]. Conversely, accurate and timely information about mpox transmission, symptoms, and prevention methods may contribute to adaptive illness representations, such as illness coherence and controllability, which predict positive coping behaviors such as adherence to preventive measures and timely health care seeking [34,35]. Applying the extended CSM to mpox-related information exposure among GBMSM can provide insights into the psychological mechanisms driving preventive intentions and behaviors. This understanding can inform the development of targeted risk communication strategies that promote adaptive coping and vaccination. These relationships remain unexplored in the context of culturally sensitive mpox risk communication and vaccination campaigns for GBMSM in the Asia-Pacific region.

Behavioral intentions have been theorized (eg, theory of planned behavior) and shown to predict actual health-seeking behaviors [36], such as COVID-19 vaccination among UK adults aged 50 to 64 years [37], human papillomavirus vaccination among Dutch girls [38], and influenza vaccination for patients with diabetes in China [39]. However, Jongen et al [40] found that an increased level of perceived risk for mpox more consistently predicted and explained the difference between high intention for mpox vaccination and actual vaccination uptake among Dutch GBMSM attending sexual health services in a later survey wave. Similarly, in a cross-sectional survey of Canadian GBMSM, those with a lower perceived susceptibility to mpox were more likely to remain unvaccinated despite being offered vaccination after attending a sexually transmitted infection (STI) clinic [41]. Therefore, mpox illness perceptions may play a stronger role in the relationship between behavioral intent and subsequent uptake for GBMSM if it increases the perceived risk of contracting mpox—this hypothesized relationship is yet to be explored [42].

Mainland China and Hong Kong play pivotal roles in the global resurgence of mpox, largely due to their extensive interconnectivity and prominence as international air travel hubs. These 2 regions present distinct policy and social environments—Beijing, as the capital of mainland China, has limited vaccine availability and a higher level of societal stigma [43], whereas Hong Kong has implemented a fully subsidized vaccine program and has generally more open societal attitudes toward sexual minority groups [44]. Both cities experience significant cross-border interaction and are major population centers yet differ markedly in their public health policies, media environments, and levels of LGBTQ+ acceptance [44]. This contrast allows for a nuanced comparison of how structural factors—such as vaccine access, stigma, and risk communication—interact to shape health behaviors among GBMSM.

### Objectives

In 2023, China reported 1712 confirmed mpox cases, with the Beijing municipality (258 cases) and Guangdong province (342 cases) accounting for more than one-third of the country’s total cases [9]. Hong Kong, despite having only 68 confirmed cases, is surrounded by the Guangdong province with millions of daily cross-border travelers and observed steady local confirmed cases from January 2024 to August 2024 [45]. Using the extended CSM framework applied through multigroup structural equation modeling (SEM), this study aimed to examine how differential exposure to mpox information and perceptual processes may influence perceived susceptibility and vaccination intentions among GBMSM in these 2 highly interconnected municipal areas.

Given the multidimensional nature of information exposure, illness perceptions, and behavioral intent, this study used SEM because it enables the simultaneous estimation of complex, interrelated pathways and mediating effects, which are essential for understanding how latent constructs—such as threat perception and personal control—link information exposure to risk perception and vaccination intent. Unlike standard regression, SEM allows for the modeling of latent variables using multiple observed indicators, providing a more accurate representation of psychosocial processes. In addition, we used multigroup SEM to directly compare these pathways between GBMSM in Hong Kong and Beijing, allowing us to test for contextual differences in the relationships among key variables.

We hypothesized that regional differences in case numbers, risk communication, vaccine program availability, and cultural values lead to different levels of mpox information exposure, illness perceptions, and subsequent perceived risk and vaccination intentions. Specifically, we propose that the content of mpox information—factual, positive, or negative—may affect vaccination intentions through distinct perceptual processes by shaping threat or control perceptions. We further anticipate that these information attributes will differentially impact perceived risk and vaccination intent and that geographical and social differences between Hong Kong and Beijing may moderate these effects based on local case burdens, information exposure, perceived susceptibility, and vaccine access.

## Methods

### Study Design and Sample

**Figure 1:**
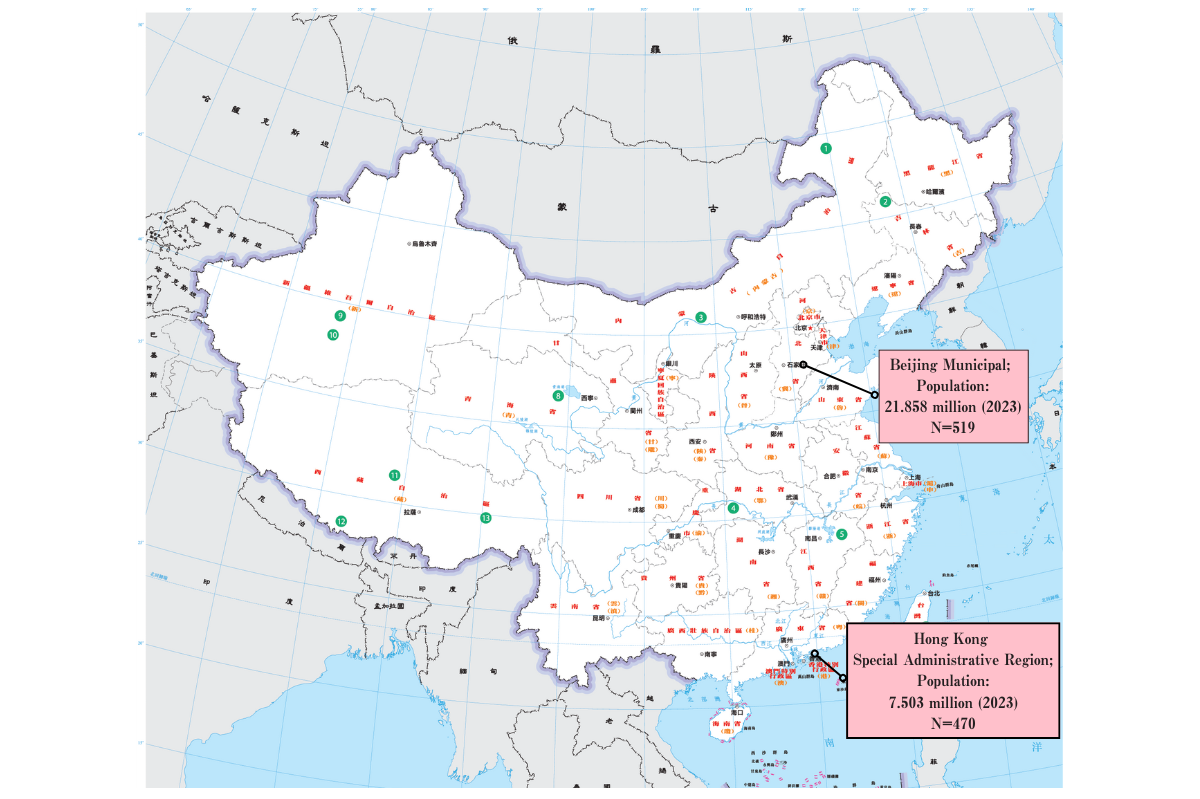
Geographical mapping of Beijing and Hong Kong with respect to gay, bisexual, and other men who have sex with men participants recruited from these municipal areas.

A cross-sectional survey was conducted with GBMSM in Beijing and Hong Kong from November 2023 to March 2024. Participants had to meet the following inclusion criteria: (1) being Chinese male individuals aged ≥18 years and (2) having engaged in anal intercourse with at least one man in the previous 6 months (geographic information and location of the study sites are presented in Figure 1). Recruitment and data collection methods were similar between Beijing and Hong Kong. In both cities, upon approval of the owners, trained and experienced field-workers approached potential participants in gay bars at different time slots on weekdays and weekends. On sites, the field-workers briefed potential participants about the study and gave them an information sheet. Online recruitment was also conducted in both cities by posting study advertisements on popular gay websites (in Hong Kong only), social media platforms such as Facebook (in Hong Kong only) or Weibo and WeChat (in Beijing only), and partner-seeking mobile apps frequently used by local GBMSM (Blued in Beijing and Grindr in Hong Kong). In Hong Kong, Facebook is the most popular social media platform, and Grindr is the most widely used gay dating app. However, these 2 online platforms are not available in mainland China. Weibo and WeChat are the 2 most commonly used social media platforms in mainland China, with functions very similar to those of Facebook. Blued is the most widely used gay dating app in mainland China, with features and functions similar to those of Grindr. Therefore, Weibo, WeChat, and Blued were chosen to facilitate online recruitment in Beijing. Potential participants who were interested in the study could contact the research team via private messaging, telephone, WhatsApp or WeChat, or email. Recruitment was supplemented through referrals made by peers and community-based organizations. Participants were screened for study eligibility. Participant anonymity was strictly maintained, and verbal informed consent was obtained. Various contact methods were used to arrange telephone interviews, which were conducted in Mandarin in Beijing and in Cantonese in Hong Kong.

### Ethical Considerations

This study was reviewed and approved by the Survey and Behavioural Research Ethics Committee of the Chinese University of Hong Kong (reference SBRE-22-0488). The research adhered to established ethical guidelines for human participant research. Verbal informed consent was obtained from all participants before taking part in the study. Participants were provided with a detailed information sheet outlining the purpose of the study, procedures, risks, and benefits before consenting. Consent was specifically obtained for the primary data collection, and no additional consent was required for the analysis as all data were anonymized.

Participant anonymity was strictly maintained throughout the study. Data collected during the phone interviews were deidentified at the point of collection, ensuring that no personally identifiable information was stored or linked to the responses. All data were securely stored in encrypted files accessible only to authorized researchers. Participants were compensated for their time upon completing the 30-minute phone interviews. In Hong Kong, participants received an HK $50 (US $6.40) supermarket coupon, and in Beijing, they were provided with a ¥20 (US $3) e-coupon. No identifiable images or personal data of individual participants were included in the manuscript or supplementary materials. As such, there is no risk of participant identification.

### Participants and Data Collection

Recruitment and data collection procedures were standardized for both Beijing and Hong Kong. Trained field-workers, with permission from venue owners, approached potential participants at various times during the week and on weekends in gay bars. The participants were given a brief description of the study and an information sheet. Online recruitment included posting advertisements on popular gay websites (in Hong Kong), social media platforms (Facebook in Hong Kong and Weibo and WeChat in Beijing), and partner-seeking apps (Blued in Beijing and Grindr in Hong Kong). Interested individuals could contact the research team via private messages, phone, WhatsApp or WeChat, or email. Peer referrals and community-based organizations also supported recruitment efforts. Participant anonymity was strictly maintained, and verbal informed consent was obtained. Various contact methods were used to arrange telephone interviews, which were conducted in Mandarin in Beijing and in Cantonese in Hong Kong. To ensure content consistency, the study questionnaire was available in simplified Chinese for Beijing and traditional Chinese for Hong Kong.

### Questionnaire Design

The questionnaire was developed by a team of public health researchers, epidemiologists, health psychologists, physicians, and representatives of nongovernmental organizations. It was then pilot-tested with 5 GBMSM, and their feedback was used to refine the final version.

### Measures

#### Background Characteristics

Participants provided information on their sociodemographic characteristics (such as age, educational level, relationship status, income, and employment status); sexual orientation; and their experiences with HIV and STI testing, HIV pre-exposure prophylaxis (PrEP), and other HIV prevention services used in the previous 12 months. They also reported HIV-related risk behaviors over the previous 6 months, including engaging in condomless anal intercourse with different types of male partners, the number of sexual partners, psychoactive substance use, and the use of drugs during sexual activity. Sexualized drug use was defined as the consumption of substances such as ketamine, methamphetamine, cocaine, cannabis, ecstasy, nonprescription hypnotics, heroin, cough suppressants, amyl nitrite (poppers), γ-hydroxybutyric acid or γ-butyrolactone, foxy, and mephedrone before or during sex.

#### Mpox-Specific Information Content Exposure

Participants were asked to rate how often they encountered information on 7 common mpox-related topics using a scale from 1 (almost never) to 4 (often). The topics were (1) mpox statistics from other countries (eg, case numbers and mortality rates); (2) mpox statistics specific to Hong Kong (eg, case numbers and mortality rates); (3) the Hong Kong government’s response to the mpox outbreak; (4) information on mpox discrimination and stigma toward LGBTQ+ individuals; (5) negative information about patients with mpox, such as severe symptoms and fatalities; (6) positive information about patients with mpox, such as recovery stories; and (7) information regarding mpox prevention and treatment. Details on the aforementioned items have been published elsewhere [22].

#### Perceived Risk of Contracting Mpox

Participants were asked the following: “Based on your current health and sexual behaviors, how do you rate your risk of contracting mpox within the following year?” (1=none; 5=very high).

#### Mpox Illness Representations

Mpox illness representations were assessed using the Brief Illness Perception Questionnaire [46], modified by asking participants to imagine a hypothetical scenario of contracting mpox and replacing “illness” with “mpox” in the original format [46]. This approach has been used in previous studies to assess illness representations of those at risk of a disease but who do not have the disease [47,48]. Participants were asked to rank each item on a Likert scale ranging from 0 to 10. These items included consequences (“If you contracted mpox, how much does your illness affect your life?”), timeline (“If you contracted mpox, how long do you think your illness will continue?”), personal control (“If you contracted mpox, how much control do you feel you have over your illness?”), treatment control (“If you contracted mpox, how much do you think existing treatment can help your illness?”), identity (“If you contracted mpox, how much do you experience symptoms from the illness?”) and concern (“If you contracted mpox, how concerned are you about your illness?”), coherence (“If you contracted mpox, how well do you feel you understand mpox?”), and emotions (“If you contacted mpox, how much does your illness affect you emotionally?”).

#### Mpox Vaccination Intent and Uptake Behaviors

Participants were asked whether they had received the mpox vaccine (yes or no) and, if not, how likely they were to receive it in the following 6 months (1=very low; 5=very high).

### Analysis

Sample characteristics were described using total row proportions, means, and SDs and by location (Beijing and Hong Kong), with differences between locations evaluated using the Pearson chi-square test or Wilcoxon rank sum test (analytic sample size: N=989; Hong Kong: n=470; Beijing: n=519) [49]. Zero-order (Pearson) correlations were computed to evaluate correlations between key variables in this study. Missing data were imputed using the nonparametric random forest algorithm [50]. Linear regression was conducted to examine the bivariate relationship between background variables and mpox vaccination intention in the following 6 months. All significant background variables in univariate regression were adjusted for in the following analyses aided by directed acyclic graphs to avoid omitted confounding effects and conditioning on a collider [49,51].

SEM was conducted to examine the hypothesized model using a 2-step approach. First, a confirmatory factor analysis was conducted to assess the goodness of fit of the measurement model. The construct reliability of the latent variables was evaluated using both the Cronbach α and McDonald ω [52]. SEM was used to test the proposed structural model. The mean and variance–adjusted weighted least squares estimator was used to estimate modeled parameters accounting for ordinally distributed endogenous outcomes [49,53]. Indirect effects were estimated using Bollen-Stine bootstrapping with 5000 repetitions, which is robust against deviation from multivariate normality corresponding to mean and variance–adjusted weighted least squares estimator and ordinal endogenous outcomes [54]. Overall model fit was determined using (1) the scaled chi-square badness-of-fit index (*P*>.05), (2) a scaled root mean square error of approximation (S-RMSEA) of <0.06, (3) the scaled comparative fit index (S-CFI), and (4) a scaled Tucker-Lewis index (S-TLI) of >0.95 [51,55,56].

Multigroup SEM was further used to examine potential differences in mpox information exposure and their associations with mpox perceptions across cities (moderation by city). We tested for partial scalar invariance in the measurement model and partial path coefficient invariance in the structural model. Invariance was determined by a difference in the S-CFI of <0.01 and a significant log-likelihood ratio test (*P*<.05) between the baseline models (ie, configural fits) and models with equality constraints on each of the latent factor loadings (weak or metric invariance) and thresholds or intercepts (strong or scalar invariance) [57,58]. The moderation of parameter estimates from the final multigroup structural equation model was examined using a 2-sided Wald test for heterogeneity using the appropriate unstandardized coefficients (*B*) and variance parameters [59], with *P*<.05 indicating a significant difference. We conducted sensitivity analyses of the direct and indirect effects of multigroup SEM by replicating the same model restricted to participants from Hong Kong and Beijing separately (Multimedia Appendix 1). Psychometric analyses were conducted using the *psych* and *lavaan* packages in R (R Foundation for Statistical Computing) [60,61].

## Results

### Sample Description

Of the eligible participants approached through outreach in gay venues, online recruitment, and peer referral (n=713 from Hong Kong and n=602 from Beijing), 86% (613/713) from Hong Kong and 87% (524/602) from Beijing provided verbal consent and completed telephone interviews. Of the original total sample (N=1137), 0.44% (5/1137) of participants from Beijing and 12.58% (143/1137) of participants from Hong Kong were vaccinated against mpox and were removed from the analytic sample. Table 1 presents the unvaccinated participant characteristics by total sample (N=989) and group (Beijing: 519/989, 52.5%; Hong Kong: 470/989, 47.5%). Among the participants from both groups, most were aged ≥25 years (859/989, 86.9%), tertiary educated (836/989, 84.5%), single (752/989, 76%), and employed full time (774/989, 78.3%); identified as homosexual (823/989, 83.2%); and earned above the median city income (612/989, 61.9%). Mpox vaccination rates were significantly higher in Hong Kong than in Beijing (116/519, 22.4% vs 4/470, 0.9%; *P*<.001); however, the average levels of vaccination intention (mean score 2.31, SD 1.24 in Beijing vs mean score 2.32, SD 1.22 in Hong Kong; *P*=.60) and perceived risk of contracting mpox (mean score 1.84, SD 0.88 in Beijing vs mean score 1.86, SD 0.82 in Hong Kong; *P*=.90) among those who were unvaccinated were not significantly different between Beijing and Hong Kong. GBMSM in Hong Kong were less likely to be married, cohabit with a woman, or identify as bisexual or other. Participants in Beijing reported a higher uptake of sexual health services, including HIV testing (440/517, 85.1% vs 185/470, 39.4%; *P*<.001) and PrEP use (100/517, 19.3% vs 39/470, 8.3%; *P*=.03), in the previous 6 months.

**Table 1:** Sample characteristics among mpox-unvaccinated gay, bisexual, and other men who have sex with men participants from Hong Kong and Beijing, China (N=989).

Characteristic				Overall		Beijing (n=519)		Hong Kong (n=470)		*P* value^a^	
**Sociodemographic characteristics, n (%)**											
	**Age group (y)**										<.001
		18-24	130 (13.1)		64 (12.3)		66 (14)				
		25-34	488 (49.3)		276 (53.2)		212 (45.1)				
		35-44	256 (25.9)		138 (26.6)		118 (25.1)				
		≥45	115 (11.6)		41 (7.9)		74 (15.7)				
	**Educational level**										.03
		Secondary or lower	153 (15.5)		68 (13.1)		85 (18.1)				
		Tertiary or higher	836 (84.5)		451 (86.9)		385 (81.9)				
	**Current relationship status**										<.001
		Currently single	752 (76)		365 (70.3)		387 (82.3)				
		Married or cohabiting with a man	199 (20.1)		119 (22.9)		80 (17)				
		Married or cohabiting with a woman	38 (3.8)		35 (6.7)		3 (0.6)				
	**Monthly income level**										<.001
		Below the city median	339 (34.3)		190 (36.6)		149 (31.7)				
		Above the city median	612 (61.9)		294 (56.6)		318 (67.7)				
		Missing	38 (3.8)		35 (6.7)		3 (0.6)				
	**Current employment status**										.20
		Full time	774 (78.3)		414 (79.8)		360 (76.6)				
		Other	215 (21.7)		105 (20.2)		110 (23.4)				
	**Sexual orientation**										<.001
		Homosexual	823 (83.2)		403 (77.6)		420 (89.4)				
		Bisexual, heterosexual, or uncertain	166 (16.8)		116 (22.4)		50 (10.6)				
**Health service use in the previous 6 mo, n (%)**											
	**HIV testing**										<.001
		No	364 (36.8)		79 (15.2)		285 (60.6)				
		Yes	625 (63.2)		440 (84.8)		185 (39.4)				
	**STI^b^ testing**										<.001
		No	509 (51.5)		187 (36)		322 (68.5)				
		Yes	480 (48.5)		332 (64)		148 (31.5)				
	**HIV pre-exposure prophylaxis use**										<.001
		No	850 (85.9)		419 (80.7)		431 (91.7)				
		Yes	139 (14.1)		100 (19.3)		39 (8.3)				
**Sexual behaviors in the previous 6 mo**											
	Number of regular sex partners, mean (SD)			1.42 (2.27)		1.71 (2.91)		1.10 (1.11)		.003	
	**Condomless anal sex with regular sex partners, n (%)**										.02
		No	534 (54)		299 (57.6)		235 (50)				
		Yes	455 (46)		220 (42.4)		235 (50)				
	Number of nonregular sex partners, mean (SD)			1.94 (5.40)		2.20 (6.73)		1.65 (3.37)		.02	
	**Condomless anal sex with nonregular sex partners, n (%)**										.02
		No	806 (81.5)		409 (78.8)		397 (84.5)				
		Yes	183 (18.5)		110 (21.2)		73 (15.5)				
	**Sexualized drug use in the previous 6 mo, n (%)**										<.001
		No	854 (86.3)		424 (81.7)		430 (91.5)				
		Yes	135 (13.7)		95 (18.3)		40 (8.5)				
**Risk perception and intention to receive mpox vaccine, mean (SD)**											
	Perceived risk of contracting mpox			1.84 (0.88)		1.83 (0.82)		1.86 (0.93)		.90	
	Mpox vaccination intent			2.31 (1.24)		2.32 (1.22)		2.29 (1.27)		.60	

^a^Pearson chi-square test or Wilcoxon rank sum test for group comparison.

^b^STI: sexually transmitted infection.

### Distribution, Construct Reliability, and Validity Among Latent Variables

Table 2 shows the means and SDs of the observed items of the latent factors from the measurement model. The average self-rating of most observed items of the latent factors was higher among participants in Beijing than among those in Hong Kong except for some mpox illness perceptions (Brief Illness Perception Questionnaire) measures. Reliability coefficients indicated adequate internal consistency for most latent factors except for the control perception domain of mpox illness perception. Thus, only 1 personal control item from mpox illness perceptions was used in subsequent structural equation models. Zero-order correlations showed high correlations among factual, positive, and negative mpox information content (Table 3). The threat and personal control perception domains of mpox illness perceptions had low to moderate correlations with other latent variables, indicating discriminant validity that was consistent across the Beijing and Hong Kong subsamples.

**Table 2:** Descriptive statistics of observed indicators and reliabilities of latent variables among mpox-unvaccinated gay, bisexual, and other men who have sex with men participants from Hong Kong and Beijing, China (N=989).

				Score range		Beijing (n=519)							Hong Kong (n=470)							*P* value^a^
						Score, mean (SD)		Cronbach α		McDonald ω		Score, mean (SD)			Cronbach α		McDonald ω			
**Frequency of exposure to information**																				
	**Factual content**							0.90		0.90					0.70		0.78			
		International mpox statistics	1-4		2.17 (0.97)						1.79 (0.89)							<.001		
		Local mpox statistics	1-4		2.21 (0.94)						1.94 (0.96)							<.001		
		Government’s responses	1-4		2.16 (0.94)						1.83 (0.88)							<.001		
	**Positive content**							0.86		0.85					0.72		0.77			
		Patients recovered from mpox	1-4		2.35 (1.00)						1.69 (0.87)							<.001		
		Prevention and treatment of mpox	1-4		2.31 (0.96)						2.10 (0.95)							<.001		
	**Negative content**							0.73		0.74					0.73		0.75			
		Discrimination and stigma against LGBTQ+^b^ individuals	1-4		2.67 (1.06)						2.46 (1.16)							.001		
		Patients with severe symptoms and deaths	1-4		2.33 (0.99)						2.10 (1.01)							<.001		
**Mpox illness perceptions (BIPQ^c^)**																				
	**Threat perceptions**							0.94		0.94					0.77		0.78			
		Consequences	0-10		7.93 (2.72)						7.35 (2.22)							<.001		
		Timeline	0-10		7.24 (2.68)						5.42 (2.26)							<.001		
		Identity	0-10		7.57 (2.57)						7.50 (1.93)							.59		
		Concern	0-10		8.20 (2.59)						7.77 (2.40)							.004		
		Emotion	0-10		7.81 (2.69)						7.78 (2.12)							.82		
	**Control perceptions**							0.60		0.61					0.33		0.54			
		Coherence^d^	0-10		5.53 (3.10)						4.92 (2.21)							<.001		
		Personal control	0-10		6.72 (2.86)						6.22 (2.21)							.001		
		Treatment control^d^	0-10		7.23 (2.74)						7.44 (1.86)							.12		

^a^*P* value from Kruskal-Wallis test for group comparison.

^b^LGBTQ+: lesbian, gay, bisexual, transgender, and queer.

^c^BIPQ: Brief Illness Perception Questionnaire.

^d^Excluded from analysis due to differential item cross-loadings across groups, contributing to poor reliability and convergent validity.

**Table 3:** Zero-order (Pearson) correlation matrix among key factors from the measurement model among total gay, bisexual, and other men who have sex with men participants (N=989) and separately for those from Beijing (n=524) and Hong Kong (n=613).

		Factual information	Positive information	Negative information	Threat perceptions	Personal control perceptions	Perceived risk of contracting mpox	Vaccination intent for mpox
**Total**								
	Factual information	1	—^a^	—	—	—	—	—
	Positive information	0.85	1	—	—	—	—	—
	Negative information	0.84	0.81	1	—	—	—	—
	Threat perceptions	0.21	*0.14* ^b^	0.31	1	—	—	—
	Personal control perceptions	0.36	*0.39*	*0.30*	0.27	1	—	—
	Perceived risk of contracting mpox	*0.05*	*0.08*	*0.08*	*0.05*	*0.03*	1	—
	Vaccination intent for mpox	0.22	0.19	0.22	0.16	*0.11*	0.27	1
**Beijing**								
	Factual information	1	—	—	—	—	—	—
	Positive information	0.85	1	—	—	—	—	—
	Negative information	0.84	0.86	1	—	—	—	—
	Threat perceptions	0.18	*0.11*	0.31	1	—	—	—
	Personal control perceptions	0.31	*0.33*	*0.28*	0.27	1	—	—
	Perceived risk of contracting mpox	*0.11*	*0.14*	*0.19*	*0.11*	−*0.003*	1	—
	Vaccination intent for mpox	0.29	0.23	0.25	0.16	*0.07*	0.27	1
**Hong Kong**								
	Factual information	1	—	—	—	—	—	—
	Positive information	0.83	1	—	—	—	—	—
	Negative information	0.86	0.78	1	—	—	—	—
	Threat perceptions	0.19	*0.07*	0.31	1	—	—	—
	Personal control perceptions	0.43	*0.49*	*0.36*	0.17	1	—	—
	Perceived risk of contracting mpox	*0.001*	*0.06*	−*0.02*	*0.01*	*0.13* ^*c*^	1	—

^a^Not applicable.

^b^Coefficients in italics signify qualitatively different variable-by-variable correlations between the total sample and the subsamples by city.

^c^Significant difference of the variable-by-variable correlation coefficient from the null value (coefficient=0).

Table S1 in Multimedia Appendix 2 shows that the bivariate relationships of having tertiary education or higher, having an income level above the city’s median income, having been tested for HIV and STIs and using PrEP, having higher numbers of regular and nonregular sex partners in the previous 6 months, and having a higher perceived risk for contracting mpox in the following 12 months were associated with higher levels of intent to vaccinate against mpox in the following 6 months. Figure S1 in Multimedia Appendix 2 shows the directed acyclic graphs of the hypothesized causal relationships to derive a minimal sufficient adjustment set for confounding control consisting of age, educational level, HIV testing, PrEP use, and number of sexual partners [62,63]. Multimedia Appendix 3 shows the Strengthening the Reporting of Observational Studies in Epidemiology statement checklist.

### SEM Results

#### Model Fit Statistics

The measurement model yielded a good fit to the data (χ^2^_124_=161.0; S-CFI=0.989; S-TLI=0.986; S-RMSEA=0.049, 90% CI 0.041-0.057; standardized root mean square residual=0.030; weighted root mean square residual=0.920). Its standardized factor loading ranged from 0.72 to 0.89 (*P*<.001 in all cases). The structural model also yielded a good fit to the data (χ^2^_356_=323.0; S-CFI=0.986; S-TLI=0.992; S-RMSEA=0.030, 90% CI 0.025-0.035; standardized root mean square residual=0.019; weighted root mean square residual=1.083).

#### Path Coefficient (Direct Effects)

**Figure 2:**
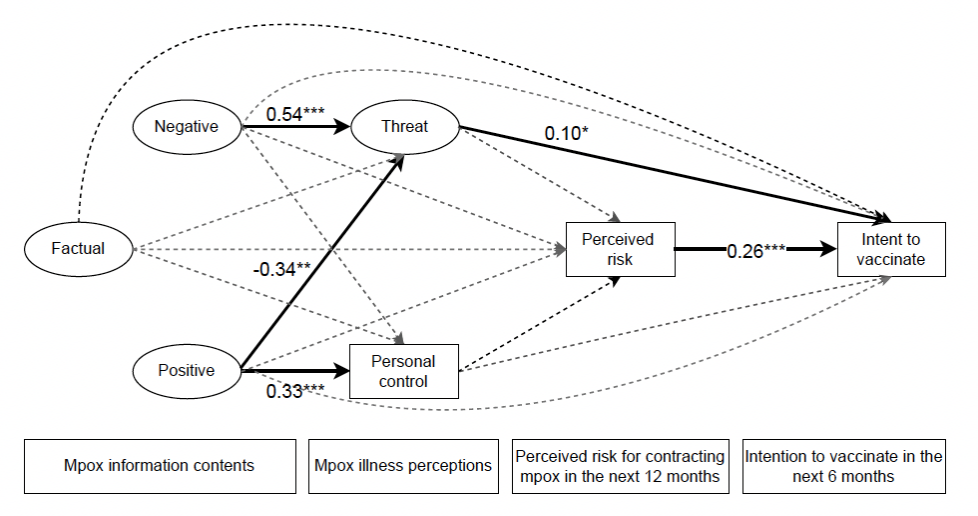
Path diagram of structural equation model with statistically significant standardized coefficients for the total mpox-unvaccinated gay, bisexual, and other men who have sex with men participants (N=989). The model was adjusted for educational level, age, HIV testing, and pre-exposure prophylaxis use; number of nonregular partners; and number of regular partners in the previous 6 months. *P<.05; **P<.01; ***P<.001.

Figure 2 shows the statistically significant path coefficients of the structural equation model combining Hong Kong and Beijing participants. Positive mpox information content (standardized path coefficient β=−0.34; *P*=.001) was negatively associated with threat perceptions, whereas negative mpox information content (β=0.54; *P*<.001) was positively associated with threat perceptions of mpox. Only positive mpox information content (β=0.33; *P*=.001) was positively associated with personal control perceptions of mpox; factual mpox information content (β=0.17; *P*<.001) was marginally associated with personal control perceptions. Neither threat perceptions nor personal control perceptions were associated with perceived risk of contracting mpox. Perceived risk of contracting mpox (β=0.27; *P*<.001) and threat perceptions (β=0.10; *P*=.01) were both positively associated with intention to receive the mpox vaccine in the following 6 months.

#### Mediated Pathways (Indirect Effects)

Threat perceptions of mpox negatively mediated (unstandardized path coefficient [B]=−0.043, 95% CI −0.82 to −0.004) the association between positive mpox information content and the intention to receive the mpox vaccine. Threat perceptions of mpox positively mediated (B=0.07, 95% CI 0.012-0.128) the association between negative mpox information content and vaccination intention.

### Multigroup Analysis and Invariance Testing

#### Overview

As indicated in Table 4, the results of the multigroup analysis of the measurement model showed that the configural model (baseline) had an acceptable fit (comparative fit index [CFI]=0.991; root mean square error of approximation [RMSEA]=0.050). The metric invariance model, with all factor loadings constrained to be equal across groups, showed a statistically meaningful decrease in model fit compared to the configural model (ΔCFI=−0.006; ΔRMSEA=0.011). However, partial scalar invariance was achieved by freeing the intercepts of the 2 indicators (ΔCFI=0.000; ΔRMSEA=−0.003). As in the SEM literature, multigroup analyses can be conducted if partial scalar invariance is supported [64-66].

**Table 4:** Measurement and structural invariance model fit statistics for multigroup structural equation models among mpox-unvaccinated gay, bisexual, and other men who have sex with men participants from Beijing and Hong Kong.

			Chi-square (*df*)		CFI^a^		RMSEA^b^		ΔChi-square per df		*P* value		ΔCFI^c^		ΔRMSEA^d^
**Measurement model**															
	Configural fit (baseline)	212.3 (96)		0.991		0.050		Reference		—^e^		—		—	
	Metric invariance	295.2 (104)		0.985		0.061		10.36		<.001		−0.006		0.011	
	Scalar invariance	398.3 (119)		0.978		0.069		8.09		<.001		−0.013		0.019	
	Partial scalar invariance	228.7 (111)		0.991		0.046		1.10		.15		0.000		−0.003	
**Full structural equation model**															
	Configural fit (baseline)	387.7 (338)		0.997		0.017		Reference		—		—		—	
	Metric invariance	443.5 (346)		0.993		0.024		6.976		<.001		−0.013		0.007	
	Scalar invariance	512.1 (399)		0.992		0.024		2.039		<.001		−0.044		0.020	
	Partial scalar invariance	414.5 (373)		0.997		0.015		0.765		.97		−0.007		0.002	

^a^CFI: comparative fit index.

^b^ΔCFI threshold of >0.01 indicates noninvariance model misfit.

^c^RMSEA: root mean square error of approximation.

^d^ΔRMSEA threshold of >0.01 indicates noninvariance model misfit.

^e^Not applicable.

**Figure 3:**
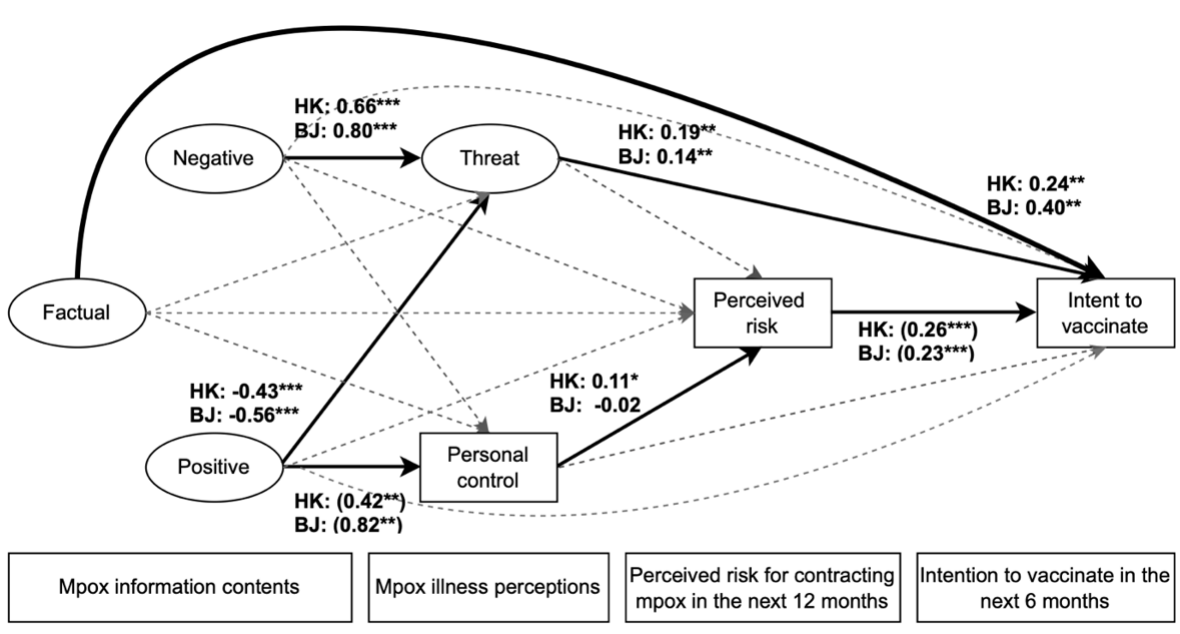
Path diagram of multigroup structural equation model with statistically significant standardized coefficients for gay, bisexual, and other men who have sex with men participants in Hong Kong (HK; n=470) and Beijing (BJ; n=519). The model was adjusted for education, age, HIV testing, and pre-exposure prophylaxis use; number of nonregular partners; and number of regular partners in the previous 6 months. Path coefficients in parentheses indicate that the coefficient parameters were estimated with equality constraints. *P<.05; **P<.01; ***P<.001.

Figure 3 shows the standardized regression path coefficients of the multigroup structural equation model. To determine whether the city moderated the paths, nested models were formed by comparing equality-constrained parameter models with the unconstrained free parameter model by city using the likelihood ratio test (chi-square≥3.8 per df as the critical value). Bolded paths indicate significant coefficients for either the Hong Kong or Beijing subsample. Estimates in parentheses indicate coefficients estimated with equality-constrained parameters and vice versa.

#### Relationships Among Attributes of Mpox Information Exposure, Threat Perceptions, Perceived Risk, and Vaccination Intent

Table 5 shows unstandardized direct and indirect effect coefficients of the relationships among attributes of mpox information exposure, threat perception, perceived risk, and vaccination intent. Exposure to positive mpox information was negatively associated with threat perceptions in Hong Kong (*B*=−0.22, 95% CI –0.38 to –0.06) and Beijing (*B*=−0.65, 95% CI –1.00 to –0.30), with a stronger association in Beijing (Wald test: *Z*=2.34 and *P*=.02). Exposure to negative information was positively associated with threat perceptions in Hong Kong (*B*=0.33, 95% CI 0.15-0.51) and Beijing (*B*=0.93, 95% CI 0.52-1.34), with a stronger association in Beijing (Wald test: *Z*=−3.40 and *P*=.002). Threat perception was positively associated with vaccination intent in Hong Kong (*B*=0.39, 95% CI 0.15-0.63) and Beijing (*B*=0.12, 95% CI 0.01-0.23), with a stronger association in Hong Kong (Wald test: *Z*=2.14 and *P*=.03). Negative information exposure had an insignificant negative association with perceived risk in Hong Kong (*B*=−0.10, 95% CI –0.30 to 0.10) and an insignificant positive association with perceived risk in Beijing (*B*=0.20, 95% CI –0.05 to 0.46), but the difference between cities was significant (Wald test: *Z*=−2.06 and *P*=.04).

**Table 5:** Unstandardized direct and indirect effect coefficients and Wald test of heterogeneity of effects by group.

		Hong Kong, *B*^a^ (SE; 95% CI)	Beijing, *B* (SE; 95% CI)	Wald test^b^	
				*Z*	*P* value
**Direct effects**					
	Positive^c^→threat^d^	−*0.22 (0.08; −0.38 to −0.06)*^e^	−*0.65 (0.18; −1.00 to −0.30)*	*2.34*	*.02*
	Negative^f^→threat	*0.33 (0.09; 0.15 to 0.51)*	*0.93 (0.21; 0.52 to 1.34)*	−*3.40*	*.002*
	Positive→risk^g^	0.14 (0.10; −0.06 to 0.34)	0.04 (0.11; −0.18 to 0.26)	0.65	.52
	Negative→risk	−0.10 (0.10; −0.30 to 0.10)	0.20 (0.13; −0.05 to 0.46)	−*2.06*	*.04*
	Threat→intent^h^	*0.39 (0.12; 0.15 to 0.63)*	*0.12 (0.06; 0.01 to 0.23)*	*2.14*	*.03*
	Personal control^i^→risk	*0.05 (0.02; 0.01 to 0.09)*	−0.02 (0.01; −0.04 to 0.01)	*3.05*	*.002*
	Personal control→intent	*0.06 (0.03; 0.001 to 0.12)*	−0.02 (0.03; −0.05 to 0.02)	*2.53*	*.01*
**Indirect effects^j^**					
	Positive→threat→intent	−*0.09 (0.04; −0.17 to −0.007)*	−0.08 (0.04; −0.16 to 0.00)	−0.13	.89
	Negative→threat→intent	*0.13 (0.05; 0.03 to 0.23)*	*0.11 (0.06; 0.002 to 0.22)*	0.19	.85
	Positive→personal control→risk→intent	*0.01 (0.01; 0.002 to 0.03)*	−0.01 (0.00; −0.01 to 0.003)	*2.52*	*.01*
	Negative→personal control→risk→intent	0.00 (0.00; −0.01 to 0.003)	0.00 (0.00; −0.002 to 0.005)	−1.20	.23
	Positive→personal control→intent	0.05 (0.03; −0.002 to 0.10)	−0.01 (0.01; −0.04 to 0.02)	*2.17*	*.03*
	Negative→personal control→intent	−0.01 (0.01; −0.04 to 0.01)	0.004 (0.01; −0.007 to 0.01)	−1.13	.26

^a^Unstandardized path coefficients.

^b^Wald test of heterogeneity of effects across groups.

^c^Positive mpox information latent variable.

^d^Threat perception latent variable composed of items from the Brief Illness Perception Questionnaire (BIPQ).

^e^Values in italics correspond to *P*<0.05.

^f^Negative mpox information latent variable.

^g^Perceived risk of contracting mpox within the following 12 months.

^h^Intent to receive the mpox vaccine in the following 6 months.

^i^Personal control item from the BIPQ.

^j^Bollen-Stine bootstrapped SEs and 95% CIs with 5000 repetitions.

#### Relationships Among Personal Control Perceptions, Perceived Risk of Contracting Mpox, and Vaccination Intent

Personal control perception was positively associated with perceived mpox risk in Hong Kong (*B*=0.05, 95% CI 0.01-0.09) and insignificantly negatively associated with perceived mpox risk in Beijing (*B*=−0.02, 95% CI –0.04 to 0.01), with a significant city difference (Wald test: *Z*=3.05 and *P*=.002). It was also positively associated with vaccination intent in Hong Kong (*B*=0.06, 95% CI 0.001-0.12) but not in Beijing (*B*=−0.02, 95% CI –0.05 to 0.02), with a significant city difference (Wald test: *Z*=2.53 and *P*=.01).

### Indirect Effects

The indirect effect of positive mpox information exposure on vaccination intent through personal control and mpox risk was significant and positive in Hong Kong (*B*=0.01, 95% CI 0.002-0.03) but not in Beijing (*B*=−0.01, 95% CI –0.01 to 0.003), with a significant difference between cities (Wald test: *Z*=2.52 and *P*=.01). The indirect effect through personal control was significant and positive in Hong Kong (*B*=0.05, 95% CI –0.002 to 0.01) but not in Beijing (*B*=−0.01, 95% CI –0.007 to 0.01), with a significant difference between cities (Wald test: *Z*=2.17 and *P*=.03).

## Discussion

### Principal Findings

Since the 2022 multicountry mpox outbreak, mpox has continued to spread in places with little access to vaccines, whereas its resurgence across countries is imminent [67]. China is home to >12 million GBMSM and has the highest mpox case burden in the Western Pacific region [4,68]. However, only 1% (5/524) of participants from China’s mpox epicenter, Beijing, had been vaccinated against mpox. To anticipate the regulatory approval of mpox vaccines in mainland China, this study examined the novel pathways through which exposure to different attributes of mpox information and perceptual processes could influence GBMSM’s vaccination decisions, specifically by raising GBMSM’s level of perceived risk of contracting mpox. Our findings provide timely implications for GBMSM-tailored risk communication and community partnerships to raise awareness of mpox among GBMSM across borders.

Our study highlights the critical roles of mpox-related information content and disease-related perceptions in shaping perceived risk and subsequent vaccine-related decision-making among GBMSM, extending the findings of Jongen et al [40]. In Hong Kong, increased exposure to positive mpox information was associated with higher personal control and vaccination intent, mediated by increased perceived risk. However, this relationship was not observed in Beijing. Factual mpox information was associated with increased vaccination intent in both cities, as demonstrated by the multigroup structural equation model. Correlation analyses also revealed that perceived risk was positively correlated with personal control perceptions in Hong Kong but not in Beijing. Vaccination intent had a stronger positive correlation with personal control in Hong Kong than in Beijing. These results suggest that, in places where fully government-sponsored mpox vaccines are in place, promoting factual and positive mpox information (eg, positive portrayal of patients with mpox, recovery stories, and health advice) could potentially increase vaccination intent by raising the awareness of their level of perceived risk of contracting mpox, which may also promote GBMSM’s personal control of mpox, which may include having accessible vaccination programs in place at GBMSM’s disposal and their related promotional campaigns enacting effective coping strategies. This finding is consistent with behavior change theories, including the extended CSM framework underlying the role of control perceptions in motivating vaccine uptake behaviors [32].

Our study shows that exposure to negative mpox information could raise GBMSM’s threat perceptions of mpox without necessarily increasing their perceived risk of contracting mpox and subsequent vaccination intent but that it is associated with increased behavioral intention for mpox vaccination. These results suggest that the association between negative mpox information and behavioral intention for mpox vaccinations through threat perceptions may be less predictive of actual vaccination behaviors as compared to positive content through personal control, which in turn increases both risk perceptions and behavioral intention to receive the mpox vaccine. Highly threatening or stigmatizing content may trigger maladaptive coping behaviors such as denial, avoidance, or disengagement [33]. Rather than motivating action, overwhelming threat can result in fatalism or psychological withdrawal, especially in communities with limited resources or structural barriers to prevention (eg, lack of vaccine access in mainland China). This finding may correspond to the high prevalence of sensationalized headlines and exaggerated reporting of severe mpox cases, mortality, and HIV or AIDS coinfection across regions [69]. This negatively charged media coverage that appeals to the public’s consumption of polarizing views and affective processes without providing risk information relevant to GBMSM, including unmoderated homophobic user comments on media outlets (homophobic slurs remain unmoderated in one of Hong Kong’s online news outlets [70]), could counter mpox control efforts [4]. Among Chinese adults exposed to disgusting graphic mpox information, those with higher disgust endorsed more avoidant preventive measures and perceived higher mpox severity but not susceptibility or perceived risk of contracting mpox [71]. In addition, higher levels of mpox-related stigma attenuate the association between perceived severity and preventive intent [71]. Emotionally provoked viewers, including GBMSM, may dissociate from their own mpox risk and view those at risk as “distant others” (eg, people living with HIV) who should be receiving the vaccine rather than themselves [72]. In addition, exposure to stigmatizing mpox information could galvanize GBMSM’s self-stigma and identity concealment, which negatively predicted mpox vaccination intent and uptake among Canadian GBMSM in the study by Le Forestier et al [8].

Despite higher levels of mpox information exposure and illness perceptions, GBMSM from Beijing were different from those from Hong Kong. This suggests that GBMSM from Beijing may not be able to identify relevant mpox risk information even with more exposure to local cases and case information. In Hong Kong, health authorities collaborate with community partners and media channels to disseminate tailored mpox risk communication and vaccination promotion for GBMSM and remove hate speech toward LGBTQ+ individuals in online news outlets [73,74]. Moreover, our finding that personal control perception more strongly predicted vaccination intention among GBMSM in Hong Kong than in Beijing may be explained by several contextual and theoretical factors. First, Hong Kong’s public health infrastructure and government-sponsored vaccination programs are widely regarded as transparent and accessible, which can enhance individuals’ trust in health authorities and the perceived efficacy of their own preventive actions. This aligns with the CSM, which posits that, when individuals believe that their actions (such as seeking vaccination) will be supported and effective within their health care environment, perceptions of personal control are more likely to translate to concrete health behaviors [32,33]. In contrast, in Beijing and mainland China, limited access to mpox vaccines, lower visibility of LGBTQ+-inclusive health campaigns, and persistent stigma may undermine individuals’ confidence that their actions will be effective or socially supported. In addition, Hong Kong’s relatively open media environment and consistent dissemination of affirmative health messages may reinforce the notion that vaccination is both accessible and beneficial, thus strengthening the link between personal control and intent. Conversely, in Beijing, mixed or negative messaging, combined with structural and social barriers, may weaken this relationship [75,76]. Thus, the interplay among health care system trust, policy transparency, and supportive media narratives likely explains why personal control perceptions are more predictive of vaccination intention in Hong Kong.

Conversely, GBMSM from mainland China may lack adequate exposure to relevant environmental risk communication. Leiwen et al [77] found that only one-fourth of mainland Chinese GBMSM correctly identified GBMSM as being at risk of contracting mpox, whereas half believed that only people living with HIV are at risk. Similarly, Zheng et al [78] found that more than half of GBMSM incorrectly identified that there were effective medications for mpox and less than half could identify >3 ways to prevent mpox infections. These findings suggest that tailored and comprehensive risk communication for GBMSM in mainland China could improve their mpox risk self-assessment and promote preventive measures even when vaccines are not within the range of options, for example, reducing the number of sexual partners and using condoms to reduce the risk of mpox transmission through direct genital contact [79]. Such communication could be synergized with positive portrayals of stories of patients with mpox, such as gay-identifying physicians and social media influencers with mpox [15,16], in raising GBMSM’s cognitive perceptions of mpox and reducing their emotional distance on perceived susceptibility while empowering them to enact preventive behaviors.

### Limitations

The results of this study should be interpreted within their limitations. First, the cross-sectional design cannot ascertain causality, specifically the temporal direction of how previous information affects subsequent intent. It is causally plausible that behavioral intent assessed at 1 point in time is a function of previous information and psychological processes. Longitudinal studies are preferred to clarify causality and the potential for reverse or bidirectional effects. Second, our recruitment strategy—using social media, gay bars, and peer referrals—may have introduced selection bias. This approach likely overrepresented younger, more digitally connected, and socially active GBMSM who are comfortable accessing online platforms or community venues while potentially underrepresenting older, less connected, or more marginalized individuals who may not participate in such spaces. As a result, the findings may not fully reflect the experiences or perspectives of the entire GBMSM population in Beijing and Hong Kong, particularly those less engaged with the LGBTQ+ community or less reachable through digital or venue-based outreach. Third, there is a possibility that unmeasured confounders influenced our study results. Factors such as previous vaccination hesitancy, underlying attitudes toward vaccination, previous experiences with health care systems, or structural barriers to health care access were not directly measured but could have independently affected both information exposure and vaccination intentions. Fourth, although our study included participants from 2 major urban centers—Beijing and Hong Kong—the generalizability of our findings to GBMSM living in smaller cities, rural areas, or other regions of China may be limited. GBMSM in less urbanized areas may have different patterns of information exposure, levels of stigma, health care access, or vaccine availability, which could influence both risk perceptions and preventive behaviors. Finally, our sample of participants from Beijing had a higher level of education, included less individuals who identified as bisexual, and had been more recently HIV tested than participants in another respondent-driven sample of GBMSM from Beijing [80]. This indicates that we were more likely to have overestimated the prevalence of behavioral intent for mpox vaccination. However, our population parameter estimate is still more conservative than those in other studies (>80%) with larger sample sizes [78,81]. On the other hand, participants from Hong Kong had similar characteristics to those of a larger, more representative sample [82]. All data were self-reported and, therefore, were subject to recall and social desirability biases. The use of trained interviewers with structured questionnaires and interview designs in this study may have reduced some reporting bias. However, questions related to sexual behavior could be stigmatized when asked by interviewers, which could have been underreported.

### Conclusions

This study highlights the critical role of mpox-related information exposure in influencing behavioral intentions toward vaccination among GBMSM in Beijing and Hong Kong. Positive information exposure, particularly regarding recovery stories and prevention, significantly increased vaccination intent by enhancing personal control perceptions and perceived risk of contracting mpox among GBMSM in Hong Kong, where a fully government-subsidized mpox vaccination program is in place. Conversely, while negative information related to mpox increased threat perceptions and vaccination intentions, it did not reliably translate to a higher perceived risk, especially in Beijing. These findings suggest several practical implications for public health messaging aimed at increasing mpox vaccine uptake among GBMSM in China. Health agencies should prioritize accurate, positively framed, and stigma-free information—such as recovery stories and practical prevention advice—delivered through channels or key opinion leaders trusted by the target community. Proactive efforts are needed to counter misinformation and stigma, for example, through myth-busting campaigns and the promotion of supportive narratives on both social and traditional media platforms. In addition to the careful moderation of stigmatizing messaging, news media channels should implement stricter standards when framing their reporting on mpox and moderate user comments on their platforms to prevent the spread of misinformation that could harm public health. Partnerships with LGBTQ+ organizations and community leaders can enhance the credibility and reach of messaging, whereas targeted outreach via social media and mobile apps can ensure that information is accessible to those most at risk. Finally, communications should provide clear guidance on accessing vaccination and related services and be culturally sensitive and inclusive to empower GBMSM to assess their risk realistically and take preventive action.

## Appendix

Multimedia Appendix 1 Sensitivity analyses with unstandardized direct and indirect effect coefficients conducted separately on gay, bisexual, and other men who have sex with men participant subsets from Hong Kong and Beijing.

Multimedia Appendix 2 Bivariate relationship between baseline characteristics and mpox vaccination intention in the following 6 months among mpox-unvaccinated gay, bisexual, and other men who have sex with men participants in Beijing and Hong Kong and directed acyclic graph representing causal relationships among variables to guide the selection of variables for statistical analyses to support causal inference.

Multimedia Appendix 3 Strengthening the Reporting of Observational Studies in Epidemiology statement—checklist of items that should be included in reports of cross-sectional studies.

## Abbreviations

CFI: comparative fit index
CSM: common sense model of illness self-regulation
GBMSM: gay, bisexual, and other men who have sex with men
LGBTQ+: lesbian, gay, bisexual, transgender, and queer
PrEP: pre-exposure prophylaxis
RMSEA: root mean square error of approximation
S-CFI: scaled comparative fit index
SEM: structural equation modeling
S-RMSEA: scaled root mean square error of approximation
STI: sexually transmitted infection
S-TLI: scaled Tucker-Lewis index

## Data Availability

The datasets generated or analyzed during this study are not publicly available because they contain sensitive personal behaviors but are available from the corresponding author on reasonable request.
