# Supplementary material for: How Information Exposure Shapes Risk Perceptions and Vaccination Intentions Among Gay, Bisexual, and Other Men Who Have Sex With Men: Cross-Sectional Survey Study
Source: JMIR Public Health Surveill. 2025 Jun 18;11:e70635. doi: 10.2196/70635 (PMC12223458; doi:10.2196/70635)
Supplement: Multimedia Appendix 1 [file publichealth_v11i1e70635_app1.docx]

Supplementary Table 3. Sensitivity analyses with unstandardized direct and indirect effect coefficients were conducted separately using gay, bisexual and other men who have sex with men participant subsets from Hong Kong and Beijing.

|  | Hong Kong | | | | Beijing | | | |
| --- | --- | --- | --- | --- | --- | --- | --- | --- |
| Direct effects | B | SE | 95% CI | | B | SE | 95% CI | |
| Positive➞Threat | -0.466 | 0.169 | -0.797 | -0.135 | **-0.639** | **0.175** | **-0.982** | **-0.296** |
| Negative➞Threat | **0.654** | **0.200** | **0.262** | **1.046** | **0.956** | **0.239** | **0.488** | **1.424** |
| Positive➞ Risk | 0.069 | 0.123 | -0.172 | 0.310 | 0.074 | 0.105 | -0.132 | 0.280 |
| Negative➞Risk | -0.061 | 0.165 | -0.384 | 0.262 | 0.161 | 0.129 | -0.092 | 0.414 |
| Threat➞Intent | **0.199** | **0.069** | **0.064** | **0.334** | **0.135** | **0.063** | **0.012** | **0.258** |
| Personal control➞ Risk | **0.110** | **0.044** | **0.024** | **0.196** | -0.059 | 0.038 | -0.133 | 0.015 |
| Personal control➞Intent | **0.119** | **0.056** | **0.009** | **0.229** | -0.061 | 0.051 | -0.161 | 0.039 |
|  |  |  |  |  |  |  |  |  |
| Indirect effects | B | SE* | 95% CI* | | B | SE* | 95% CI* | |
| Positive➞Threat➞Intent | **-0.093** | **0.483** | **-0.734** | **-0.016** | -0.086 | 2.752 | -1.436 | 0.008 |
| Negative➞Threat➞Intent | **0.130** | **4.528** | **0.026** | **2.005** | 0.129 | 4.094 | -0.011 | 2.225 |
| Positive➞Personal control➞Risk➞Intent | **0.023** | **0.608** | **0.002** | **0.109** | -0.004 | 4.402 | -0.088 | 0.015 |
| Negative➞Personal control➞Risk➞Intent | -0.009 | 1.996 | -0.442 | 0.005 | 0.001 | 6.106 | -0.011 | 1.565 |
| Positive➞Personal control➞Intent | 0.064 | 0.696 | -0.020 | 0.265 | -0.014 | 4.598 | -1.289 | 0.015 |
| Negative➞Personal control➞Intent | -0.025 | 2.365 | -0.347 | 0.020 | 0.005 | 6.371 | -0.027 | 1.150 |

Positive: positive mpox information latent variable

Negative: positive mpox information latent variable

Threat: threat perception latent variable composed of items from Brief Illness Perception Questionnaire

Person control: personal control item from Brief Illness Perception Questionnaire

Risk: Perceived risk for contracting mpox within the following 12 months

Intent: intent to vaccinate against mpox in the following 6 months

B: unstandardized path coefficients

*Bollen-Stine bootstrapped standard errors and 95% confidence intervals with 5000 repetitions.
