# Supplementary material for: How Information Exposure Shapes Risk Perceptions and Vaccination Intentions Among Gay, Bisexual, and Other Men Who Have Sex With Men: Cross-Sectional Survey Study
Source: JMIR Public Health Surveill. 2025 Jun 18;11:e70635. doi: 10.2196/70635 (PMC12223458; doi:10.2196/70635)
Supplement: Multimedia Appendix 2 [file publichealth_v11i1e70635_app2.docx]

Supplementary Table 1. Bivariate relationship between baseline characteristics and mpox vaccination intention in the next 6 months among mpox unvaccinated gay, bisexual and other men who have sex with men participants in Beijing and Hong Kong.

| **Characteristic** | **Beta** | **95% CI** | **P-value** |
| --- | --- | --- | --- |
| City |  |  |  |
| Beijing | Ref |  |  |
| Hong Kong | -0.02 | -0.18, 0.13 | 0.76 |
| Age |  |  |  |
| 18-24 | Ref |  |  |
| 25 or above | -0.01 | -0.17, 0.15 | 0.89 |
| Education level |  |  |  |
| Secondary or below | Ref |  |  |
| Tertiary or above | **0.28** | **0.06, 0.49** | **0.011** |
| Relationship status |  |  |  |
| Currently single | 0.00 | -0.19, 0.18 | 0.97 |
| Married or cohabiting with a man or women | Ref |  |  |
| Income level |  |  |  |
| Below city median | Ref |  |  |
| Above city median | **0.18** | **0.01, 0.34** | **0.033** |
| Employment status |  |  |  |
| Full time | 0.05 | -0.13, 0.24 | 0.57 |
| Others | Ref |  |  |
| Sexual orientation |  |  |  |
| Homosexual | Ref |  |  |
| Bisexual/heterosexual or uncertain | -0.17 | -0.38, 0.03 | 0.10 |
| *Health services utilization in the past 6 months* |  |  |  |
| HIV testing |  |  |  |
| No | Ref |  |  |
| Yes | **0.41** | **0.25, 0.57** | **<0.001** |
| STI testing |  |  |  |
| No | Ref |  |  |
| Yes | **0.32** | **0.16, 0.47** | **<0.001** |
| HIV preexposure prophylaxis use |  |  |  |
| No | Ref |  |  |
| Yes | **0.4** | **0.17, 0.62** | **<0.001** |
| Sexual risk behaviors in the past 6 months |  |  |  |
| Number of regular sex partners, mean (SD) | **0.03** | **0.02, 0.04** | **<0.001** |
| Condomless anal sex with regular sex partners |  |  |  |
| No | Ref |  |  |
| Yes | 0.06 | -0.10, 0.21 | 0.49 |
| Number of non-regular sex partners, mean (SD) | **0.03** | **0.02, 0.04** | **<0.001** |
| Condomless anal sex with non-regular sex partners |  |  |  |
| No | Ref |  |  |
| Yes | 0.04 | -0.16, 0.24 | 0.70 |
| Sexualized drug use |  |  |  |
| No | Ref |  |  |
| Yes | 0.13 | -0.10, 0.35 | 0.28 |
| Perceived risk for mpox, mean (SD) | **0.42** | **0.34, 0.51** | **<0.001** |

| 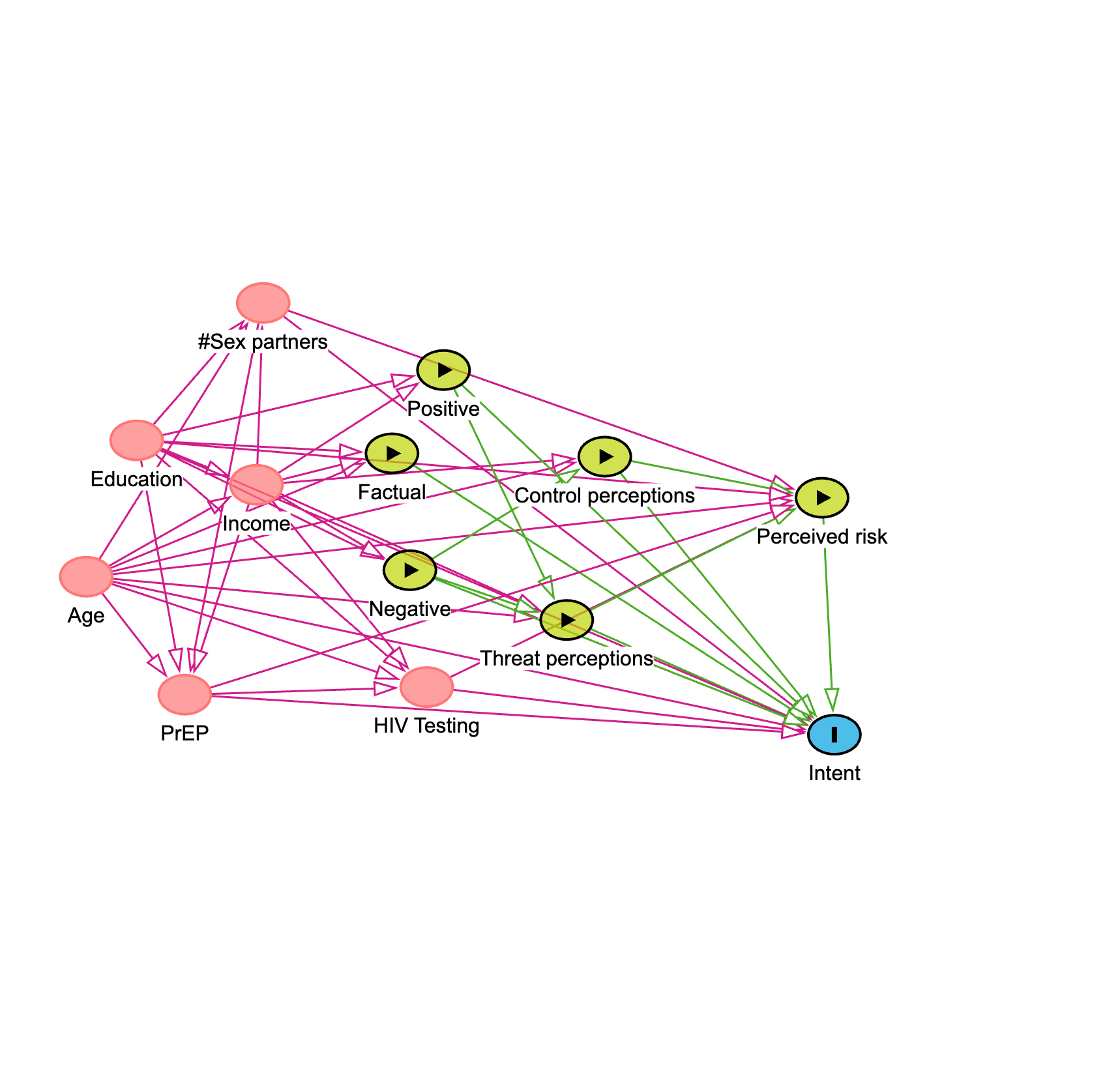  Supplementary Figure 1. Directed acyclic graph (DAG) representing causal relationships among variables to guide the selection of variable for statistical analyses to support casual inference. |
| --- |
